# Direct synthesis of urea from carbon dioxide and ammonia

**DOI:** 10.1038/s41467-023-40351-5

**Published:** 2023-07-31

**Authors:** Jie Ding, Runping Ye, Yanghe Fu, Yiming He, Ye Wu, Yulong Zhang, Qin Zhong, Harold H. Kung, Maohong Fan

**Affiliations:** 1 School of Chemistry and Chemical Engineering, Nanjing University of Science and Technology, Nanjing, Jiangsu 210094 P. R. China; 2 College of Engineering and Applied Sciences, and School of Energy Resources, University of Wyoming, Laramie, WY 82071 USA; 3 Key Laboratory of Jiangxi Province for Environment and Energy Catalysis, Institute of Applied Chemistry, College of Chemistry, Nanchang University, Nanchang, Jiangxi 330031 P. R. China; 4 Key Laboratory of the Ministry of Education for Advanced Catalysis Materials, Institute of Physical Chemistry, Zhejiang Normal University, Jinhua, Zhejiang 321004 P. R. China; 5 Department of Materials Physics, Zhejiang Normal University, Jinhua, Zhejiang 321004 P. R. China; 6 College of Chemistry and Chemical Engineering, Henan Polytechnic University, Jiaozuo, Henan 454000 PR China; 7 Department of Chemical and Biological Engineering, Northwestern University, Evanston, IL 60208 USA; 8 School of Civil and Environmental Engineering, Georgia Institute of Technology, Atlanta, GA 30332 USA

**Keywords:** Heterogeneous catalysis, Sustainability, Catalyst synthesis

## Abstract

Urea, a crucial nitrogen fertilizer, plays a vital role in meeting global food demand. However, its current production method is energy-intensive and environmentally unfriendly. In this commentary article, the authors propose strategies to address and overcome these challenges.

## Teaser

Urea is an essential fertilizer needed to meet the global demand for food. Currently, its production rate by reaction of carbon dioxide with ammonia is slow and the energy demand is high. Here we discuss strategies to overcome these challenges.

**Fig. 1:**
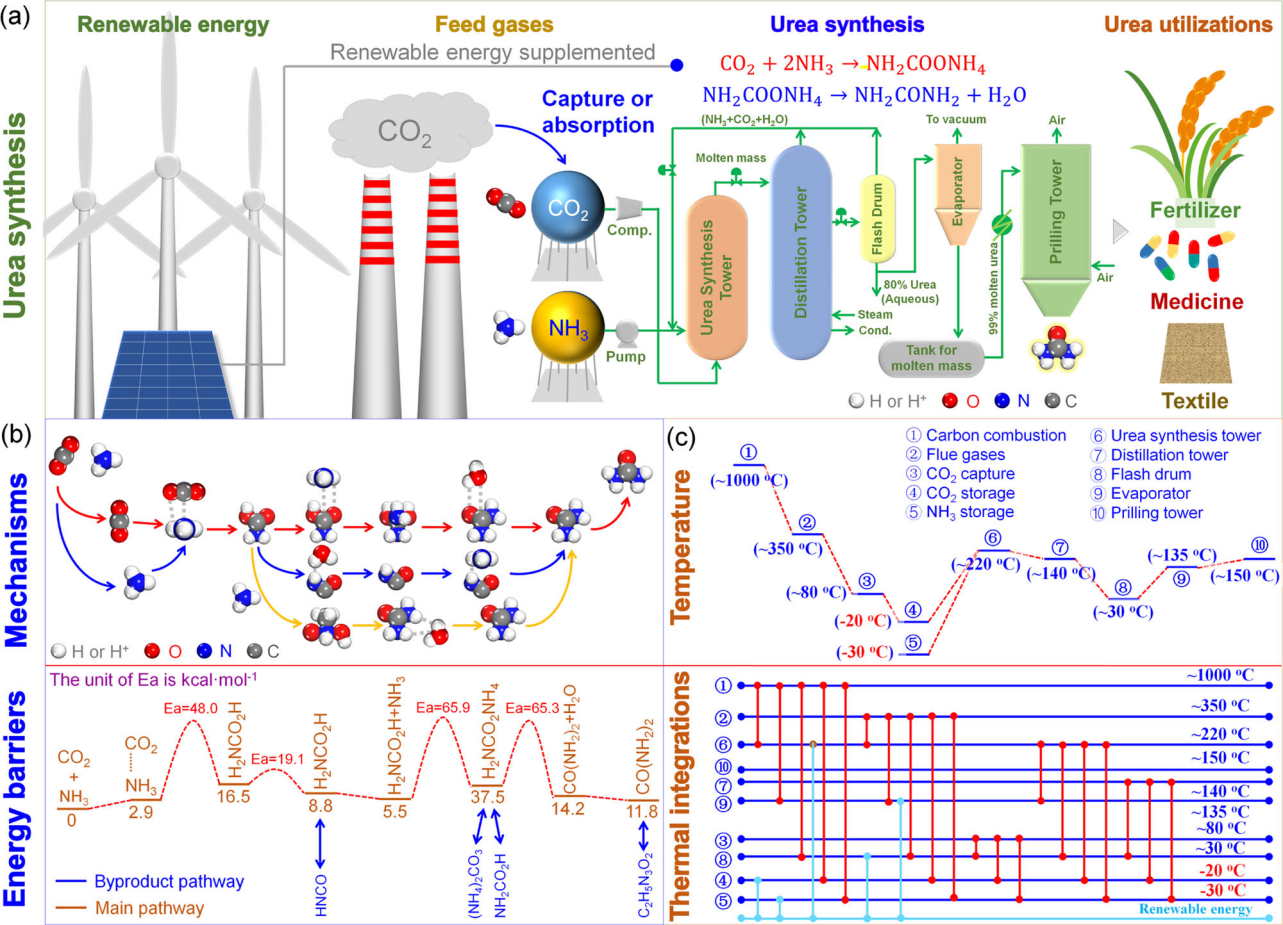
Urea synthesis process from carbon dioxide and ammonia and the challenges and strategies to achieve high production rate with low carbon intensity and energy demand. **a** Urea synthesis process using carbon dioxide captured from flue gases and ammonia synthesized using renewable energy. **b** Mechanisms of converting carbon dioxide and ammonia on the left, through a series of surface reaction intermediates, to urea on the right; the energy profile, shown in the lower portion of the panel, identifies a major energy barrier in the formation of a carbon–nitrogen bond between ammonia and carbamate. **c** Sensible heat available at each step of the synthesis process and a potential process heat integration scheme.

Urea is a vital nitrogen fertilizer that serves as the “food of food.” It plays a crucial role in meeting the rising demand for food from an expanding population. The current method for urea production uses the Bazarov reaction (Fig. 1a), in which carbon dioxide (CO_2_) and ammonia (NH_3_) are first converted to ammonium carbamate (Reaction (1)) which is then dehydrated to urea (Reaction 2)^1^.

1 $${{{{{{\rm{CO}}}}}}}_{2}+{2{{{{{\rm{NH}}}}}}}_{3}\to {{{{{{\rm{NH}}}}}}}_{2}{{{{{\rm{COO}}}}}}{{{{{{\rm{NH}}}}}}}_{4}$$

2 $${{{{{{\rm{NH}}}}}}}_{2}{{{{{\rm{COO}}}}}}{{{{{{\rm{NH}}}}}}}_{4}\to {{{{{{\rm{NH}}}}}}}_{2}{{{{{\rm{CO}}}}}}{{{{{{\rm{NH}}}}}}}_{2}+{{{{{{\rm{H}}}}}}}_{2}{{{{{\rm{O}}}}}}$$

Reaction 1 is strongly exothermic that occurs in the urea synthesis tower, where rapid removal of reaction heat is needed at a pressure high enough to liquefy ammonia and carbon dioxide. Reaction 2, which occurs in the prilling tower, is slow and slightly endothermic, and this reaction typically limits the yield of urea. Even at the severe conditions of 12.5–25.0 MPa at 170–220 ^o^C employed in a typical commercial operation, the single pass yield of urea is low (≤10%), but the carbon intensity is high due to the high energy demand (≥24.8 GJ/t urea)^2^. The slow rate of Reaction 2 is due to the kinetic barriers in forming the second carbon–nitrogen bond in urea while breaking the carbon–oxygen bond of the carbamate (Fig. 1b)^3^. A number of catalysts have been reported to facilitate this step, including water and ammonia^4^ and various soluble metal complexes and cerium dioxide^5–7^. For example, Hanson et al. developed a copper(II) sulfate complex that lowers the energy barrier of ammonium carbamate dehydration to urea by over 50%^7^. Lowering the carbon intensity and energy demand can be achieved by optimizing the thermal integration of the processing plant and utilization of renewable energy (Fig. 1c).

## Strategies for improving catalyst design for urea synthesis

The slow Reaction 2 involves bringing together a Lewis base (ammonia) and an electron-rich carbamate ion, forming a bond between the nitrogen atom of ammonia that has an electron lone pair and the electron-deficient carbon atom of the carboxylate ion, and cleaving a carbon–oxygen bond in the carbamate. Facilitating these steps requires different catalyst properties. For example, the carbon–oxygen bond cleavage step could be facilitated by a Lewis acid interacting with the oxygen atom via its electron lone pair in conjunction with hydroxyl groups that facilitate water formation, the coupling of ammonia with carbamate step by a Lewis acid that binds ammonia and enhances its surface concentration and a Lewis acid that adsorbs and increases the concentration of carbamate. Thus, an optimal catalyst is likely to be multifunctional. Indeed, frustrated Lewis acid/base pairs are reported to be effective catalysts in related reactions^8,9^. These sites need to be situated in close proximity to effect the carbon–nitrogen coupling reaction efficiently. It is also likely that the structure of the catalyst pores and the hydrogen bonding network within them, which affect solvation stabilization of the ionic intermediates, also influence the reaction rate. Indeed, such pore structure effects have been reported^10^. Nonetheless, this limited understanding of the reaction is insufficient to formulate a coherent and comprehensive strategy to improve the process.

Efficient development of the necessary multifunctional catalysts can benefit greatly from detailed knowledge of the reaction at the molecular and atomic levels. At present, there are few techniques that are capable of in-situ monitoring of the catalytic activation of carbon dioxide and ammonia, following the transformation of the reaction intermediates, and quantifying the interaction between these intermediates and the catalytic sites that include Lewis acid or base sites, oxygen vacancies and low-valence metal/non-metal atoms. In particular, there is a lack of information on the evolution of reaction intermediates leading to carbon–nitrogen coupling and carbon–oxygen cleavage^9,11^. Whereas quantum chemical modeling and computation offer insights into the energetics of possible carbon dioxide-to-urea mechanisms, the state-of-the-art methods suffer from model precision and calculation strategy availability^12^. More accurate models and calculations should include many atoms and simultaneously consider reaction temperature and reactant diffusion rate which, however, demands expensive computational resources.

**Fig. 2:**
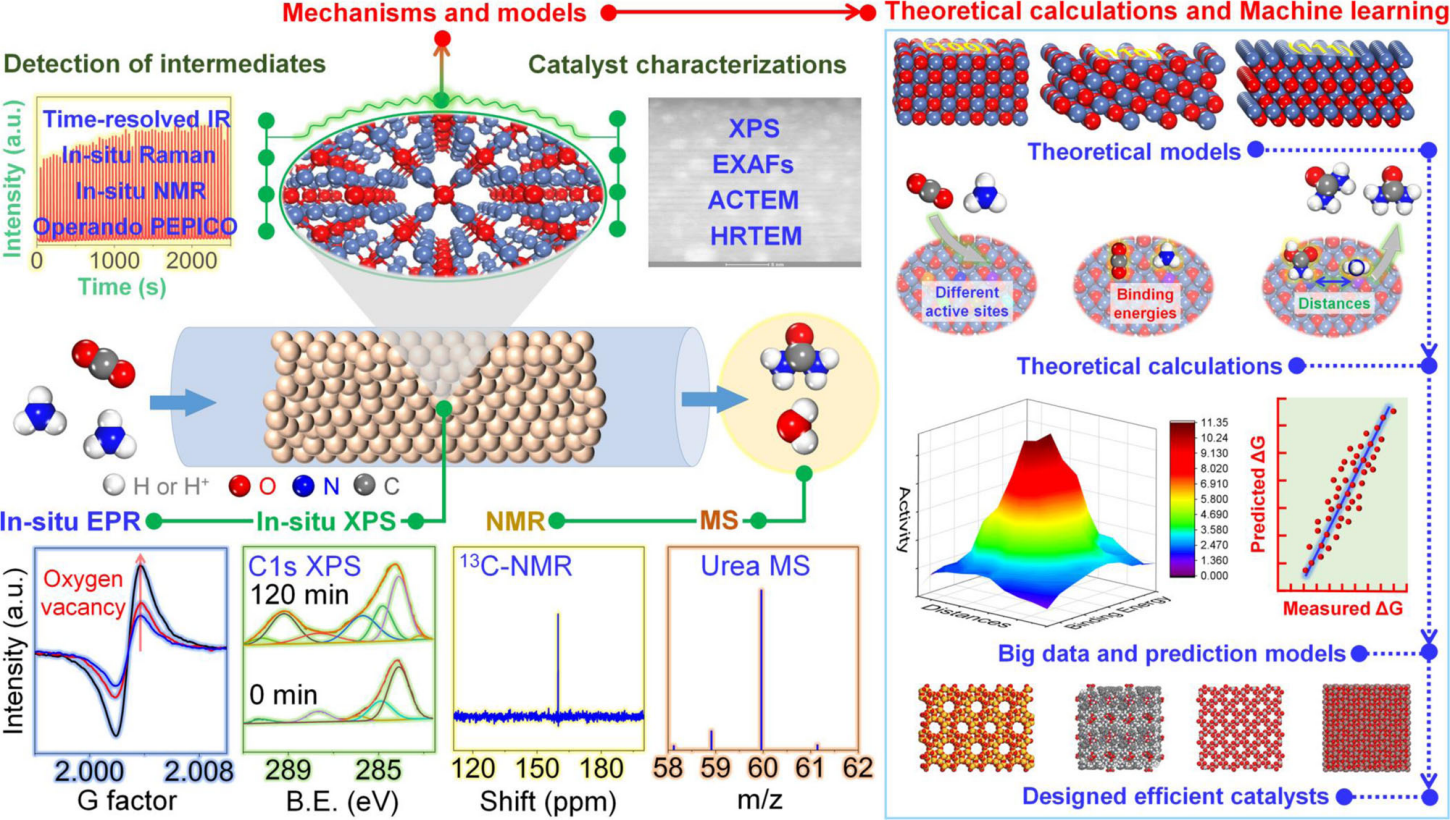
Integrations of characterization, theoretical calculation, and machine learning in catalyst design. On the left, characterization includes various spectroscopic and microscopy techniques to garner information on surface and near-surface chemical composition and structure of the working catalyst and reactive surface intermediates, kinetics, reaction rates, and product selectivities. On the right, theoretical calculations for reaction potential energy surfaces, transition state information, and energy of surface intermediates; and machine learning to identify descriptors and scaling factors for catalyst structure-property relationships. Integration and feedback between these two panels are essential to advance catalyst design (data of EPR^18^, XPS^18^, NMR^7^, MS^7^, and prediction models^16^ are from the literature).

Integrating theoretical calculations with various in-situ/operando characterizations can be a powerful tool for gaining fundamental insights (Fig. 2). However, this approach has not been used extensively in the design of carbon dioxide-to-urea catalysts, in contrast to other systems such as catalytic alcohol condensation and methanol-to-hydrocarbon reactions^13,14^. For example, Fan and coworkers combined theoretical calculations and in-situ XRD, together with AC-TEM, EXAFS^15^ to show the migration of Ag atoms into hollandite MnO_2_ (HMO) lattice tunnels to form the “pearl necklace” single-atom Ag configuration and the formation of Ag–O–Mn linkages, and deduced that the latter is essential for the oxidative acetalization of ethanol to diethoxyethane. Weckhuysen and coworkers probed the methanol-to-hydrocarbon mechanisms over H-SAPO-34 by integrating solid-state NMR and operando UV–Vis–DRS with an online MS^13^. They found that olefins, arenes, and alkanes are formed via a direct carbon–carbon bond-forming route. Pérez-Ramírez and coworkers developed a new operando photoelectron photoion coincidence (PEPICO) spectroscopy for isomer-selective identification^14^. When used together with EPR for radical identification, time-resolved spectroscopic techniques for detections of short-lived active intermediates, and theoretical calculations, they provided direct experimental evidence of methyl radicals and critical active intermediates in the methanol-to-hydrocarbons reaction.

Design of efficient catalysts for the CO_2_-to-urea reaction can benefit from such a combination of techniques, which can also be used to elucidate reaction mechanisms (Fig. 2). For example, a combination of in-situ EPR and XPS can monitor the changes of oxygen vacancies and low-valence metal/non-metal sites resulted from carbon dioxide (ammonia) chemisorption and activation. Theoretical calculations with models based on parameters from characterization can inform the electron density changes in orbitals and reaction pathways to understand these changes in the catalysts during the reaction. For example, information can be generated on changes in the electronic structure and electron density during activation of carbon dioxide and ammonia on Lewis acid or base sites, on electron donation from occupied π orbitals of a carbon dioxide molecule to empty d orbitals of the Lewis acid centers, as well as electron transfer from the Lewis base sites to the empty σ* orbital of the carbon dioxide molecule in its activation.

Structural details of stable reaction intermediates such as *CO_2_, *NH_3_, and *H_2_NCOOH (or –COO^−^) (* denotes surface adsorbed species) and their identity that is important for accurate computational modeling can be derived with a high degree of certainty by combining in-situ IR, Raman, XPS, and NMR spectroscopies. This is especially useful when the intermediates exhibit similar spectroscopic signatures with one technique but not another, such as the C = O stretching IR vibrations in *H_2_NCOOH and *H_2_NCOONH_4_. When applied in transient experiments using isotopically labeled molecules, these techniques offer valuable information on the reaction mechanism and dynamic response of the surface intermediates under reaction conditions. For short-lived active or isomeric intermediates in carbon–nitrogen coupling and carbon–oxygen bond cleavage steps, time-resolved characterization, and operando PEPICO can be very informative. For example, it is unclear whether the intermediate H_2_NCOONH_4_ directly dehydrates to urea or dehydrates to *NHCO and then couples with NH_3_ to form urea. Time-resolved spectroscopy with picosecond resolution should be very valuable in answering such questions.

## Machine learning-based catalyst design and process thermal integration

Various properties need to be optimized to achieve the most efficient multifunctional catalyst for urea synthesis, including binding and activation of the reaction intermediates as dictated by the chemical nature and atomic structure of the active sites, location of these active sites, and pore structures. Achieving these with educated trial and error experimentation can be extremely labor-intensive and time-consuming. Fortunately, cutting-edge computational and design techniques, especially machine learning, have emerged as transformative methods in the new century. Some researchers have applied machine learning methods coupled with robotic experimentation with high-throughput computation in organic synthesis to create statistical models consisting of two-dimensional descriptors with catalyst parameters and the change of Gibbs free energy for the predictive design of new reactions and catalysts^16,17^. In applying this approach to urea synthesis, one vision is to first create mechanistic models for reactions of carbon dioxide and ammonia using integrated characterization results. These models will cover various systems based on different active sites with varying strengths of Lewis acidity and basicity as well as different separation distances between carbon-based intermediates and nitrogen (oxygen)-based intermediates. Subsequently, density functional theory simulations will calculate the adsorption energies of reactants and the potential energy surfaces for the reactant activation, carbon–nitrogen coupling, and carbon–oxygen bond cleavage steps. These machine-generated data, along with published data for the elementary reactions, will form a comprehensive data set. Machine learning models will then be trained to predict the binding and dissociation energies between coupling intermediates and active sites, and the energetics of the coupling reaction. Finally, these results will be used to identify the most suitable catalysts.

At the process level, in addition to optimizing energy consumption within a carbon dioxide-to-urea plant, it may be possible to couple urea synthesis with other industrial units, such as thermal or petrochemical plants, to achieve low-energy consumption (Fig. 1c). These industries include various unit operations that contain many exothermic and endothermic processes and the heat involved can be utilized for carbon dioxide-to-urea production. For instance, one can use the hot (about 350 ^o^C) coal-fired flue gas to preheat the feed gases for carbon dioxide-to-urea production, such as for amine evaporation. The heat generated from the exothermic reaction between carbon dioxide and ammonia can be quickly removed by low-pressure steam, which is used in the bottom reboiler of the urea distillation tower. In addition, renewable energy sources, such as solar and wind power, can be used as supplementary resources. These approaches show great promise for carbon dioxide–ammonia-based urea synthesis technologies with low energy consumption and carbon emissions.
